# Older Compared With Younger Adults Performed 467 Fewer Sit-to-Stand Trials, Accompanied by Small Changes in Muscle Activation and Voluntary Force

**DOI:** 10.3389/fnagi.2021.679282

**Published:** 2021-06-21

**Authors:** Paulo Cezar Rocha dos Santos, Claudine J. C. Lamoth, Lilian Teresa Bucken Gobbi, Inge Zijdewind, Fabio Augusto Barbieri, Tibor Hortobágyi

**Affiliations:** ^1^Department of Human Movement Sciences, University Medical Center Groningen, University of Groningen, Groningen, Netherlands; ^2^Graduate Program in Movement Sciences, Posture and Gait Studies Laboratory (LEPLO), Institute of Biosciences, São Paulo State University (UNESP), Rio Claro, Brazil; ^3^Department of Biomedical Sciences of Cells and Systems, University Medical Center Groningen, University of Groningen, Groningen, Netherlands; ^4^Department of Physical Education, Graduate Program in Movement Sciences, Human Movement Research Laboratory, São Paulo State University (UNESP), Bauru, Brazil; ^5^Department of Sport Biology, Institute of Sport Sciences and Physical Education, Faculty of Sciences, University of Pécs, Pécs, Hungary; ^6^Somogy County Kaposi Mór Teaching Hospital, Kaposvár, Hungary

**Keywords:** fatigue, electromyography - EMG, functionality, muscle strength, aging

## Abstract

**Background:** Repetitive sit-to-stand (rSTS) is a fatigue perturbation model to examine the age-effects on adaptability in posture and gait, yet the age-effects on muscle activation during rSTS *per se* are unclear. We examined the effects of age and exhaustive rSTS on muscle activation magnitude, onset, and duration during ascent and descent phases of the STS task.

**Methods:** Healthy older (*n* = 12) and younger (*n* = 11) adults performed rSTS, at a controlled frequency dictated by a metronome (2 s for cycle), to failure or for 30 min. We assessed muscle activation magnitude, onset, and duration of plantar flexors, dorsiflexors, knee flexors, knee extensors, and hip stabilizers during the initial and late stages of rSTS. Before and after rSTS, we measured maximal voluntary isometric knee extension force, and rate of perceived exertion, which was also recorded during rSTS task.

**Results:** Older vs. younger adults generated 35% lower maximum voluntary isometric knee extension force. During the initial stage of rSTS, older vs. younger adults activated the dorsiflexor 60% higher, all 5 muscle groups 37% longer, and the hip stabilizers 80% earlier. Older vs. younger adults completed 467 fewer STS trials and, at failure, their rate of perceived exertion was ~17 of 20 on the Borg scale. At the end of the rSTS, maximum voluntary isometric knee extension force decreased 16% similarly in older and younger, as well as the similar age groups decline in activation of the dorsiflexor and knee extensor muscles (all *p* < 0.05).

**Conclusion:** By performing 467 fewer STS trials, older adults minimized the potential effects of fatigability on muscle activation, voluntary force, and motor function. Such a sparing effect may explain the minimal changes in gait after rSTS reported in previous studies, suggesting a limited scope of this perturbation model to probe age-effects on muscle adaptation in functional tasks.

## Introduction

Rising from and sitting down in a chair is a demanding task, as it requires high levels of knee joint torques, substantial joint excursions, inter-joint coordination, and balance (Hortobágyi et al., 2003; Netz et al., 2004; Alcazar et al., 2018; Jeon et al., 2019). Decreases in muscle strength and posture control make it especially challenging for older adults to execute the sit-to-stand task (STS) (Hurley et al., 2016; Bryanton and Bilodeau, 2017). During the STS task, older adults, in particular, tend to strongly activate the knee (e.g., rectus femoris - RF, vastus lateralis - VL, biceps femoris - BF), ankle (e.g. tibialis anterior – TA, gastrocnemius - GAS), and hip muscles (e.g., gluteus medius - Glu) (Hortobágyi et al., 2003; Jang and Yoo, 2015; Bryanton and Bilodeau, 2017, 2019; van der Kruk et al., 2021) to compensate the deficits in muscles strength (van der Kruk et al., 2021). The high mechanical load at the knee and the strong activation of the knee extensors may require some older adults to use up to 95% of the maximal capacity compared to 30–50% in younger adults (Hortobágyi et al., 2003; Bryanton and Bilodeau, 2017, 2019). Presumably, the age-related atrophy necessitates the activation of a larger portion of the available motor units, resulting in over-activation (i.e., a greater than expected muscle activation observed in younger adults). Indeed, in a number of activities of daily living, not only do the prime agonists but the antagonists also become over-activated resulting in the age-typical agonist-antagonist co-activation (Hortobágyi et al., 2003; Bautmans et al., 2011; Bryanton and Bilodeau, 2017; Chandran et al., 2019). Such an activation pattern is also associated with impaired excitatory and inhibitory control of muscle contraction and relaxation (Motawar et al., 2016) that can reduce power generation in old age (Clark et al., 2010). Imaging and magnetic brain stimulation studies suggest that age-specific imbalances in excitatory-inhibitory corticospinal circuits and cortical over-activation can accompany higher muscle activation and delay muscle relaxation (Hortobágyi and Devita, 2006; Motawar et al., 2016). It is thus conceivable that there are also age-differences in muscle activation during the performance of the STS so that both agonist and antagonist muscle groups become over- and co-activated for a longer period, increasing energy expenditure (Hortobagyi et al., 2011), underlying higher muscle fatigability in older adults (Petrella et al., 2005).

Such age-differences in muscle activation during STS may, however, not fully represent the age-effects reported previously because the cadence of STS was not controlled (Hurley et al., 2016; Bryanton and Bilodeau, 2019) or subjects executed the STS task at a very slow rate, i.e., 2 s ascent and 2 s descent (Bryanton and Bilodeau, 2017). A new element in the present compared with previous studies (Bryanton and Bilodeau, 2017) is the execution of the STS movement faster, paced by a metronome (2 s for ascent and descent). Fast STS pace would allow us to more accurately assess age-related muscle adaptations to fatigue induced by repetitive STS (rSTS). Another novelty of the present study is the examination of the effects of age on muscle activity during the descent phase and on the temporal signatures of muscle activation. Whereas, examining the descent phase during rSTS is particularly relevant to understand the age-related adaptations of eccentric force during a high demanding functional task, temporal features of muscle activity might provide new information related to age-effects on the control of muscle contraction and relaxation. Particularly, although eccentric force-producing capacity is relatively preserved, yet the control of eccentric force generation is associated with higher force variability in older compared with younger adults (Hortobagyi et al., 2001). Especially relevant, greater activation in higher-order cortical areas was described during eccentric vs. concentric force, mainly in older adults (Yao et al., 2014), which might implicate an age-specific adaption of the control of eccentric force during motor tasks. Such observation would suggest a phase- and age-specific adaptation to rSTS.

Healthy aging affects motor adaptability, i.e., flexibility in the responses to sensory and mechanical perturbations (Sosnoff and Newell, 2008). A number of perturbation paradigms have been used to determine age-effects on motor adaptability, including platform translations, split-belt, slipping, tripping, and fatigue perturbations (Liu and Lockhart, 2009; Hatton et al., 2013; Monjo et al., 2015; Santos et al., 2019; Vervoort et al., 2019). Even though studies have also used the rSTS task as a fatigue perturbation to examine age-effects on the subsequent adaptability of functional tasks, such as gait and posture (Helbostad et al., 2007; Hatton et al., 2013; Santos et al., 2019), a characterization of muscle activation during rSTS task *per se* is lacking (Bryanton and Bilodeau, 2017). A better understanding of age-differences in muscle activation during rSTS is important because these studies (Helbostad et al., 2007; Hatton et al., 2013; Santos et al., 2019) argued that changes in muscle activation due to rSTS caused adaptations in gait and posture following bouts of rSTS. However, these studies did not measure muscle activation during rSTS, nor was EMG activity measured in the target task after the rSTS perturbation. A typical compensatory strategy to submaximal fatiguing tasks involves an increase in muscle amplitude in the primary and recruitment of additional less involved muscles (Bryanton and Bilodeau, 2017, 2019). An examination of the hip, knee, and ankle muscle activation profiles, mainly assessing potential age-specific differences in ascent and descending phases, during rSTS could provide new insights into age-related motor adaptability to fatigability. Therefore, the first aim of this study was to determine the effects of age on muscle activation magnitude, onset, and duration during the ascent and descent phases of the STS performed at a controlled fast cadence (2 s for the entire STS cycle dictated by a metronome). In view of the extant data, we hypothesized that older compared with younger adults would execute the STS task with a higher, earlier, and longer-lasting muscle activation duration. We also expected phase- and age-dependent variation in muscle activation (Hortobágyi et al., 2003; Tracy and Enoka, 2006). Secondly, this study aimed to determine the effects of age and fatigue induced by rSTS on muscle activation during the initial and late-stages of STS. Given existing literature (Bryanton and Bilodeau, 2019), we expected an increase in muscle amplitude in the primary agonist knee extensors and in muscles that show low activation at the start of the STS series. Because of the age-typical neuromuscular decline (Gross et al., 1998; Hortobágyi et al., 2003; Hurley et al., 2016; Bryanton and Bilodeau, 2017), we expected a compensatory age-specific increases in muscle activation amplitude and duration after the execution of rests.

## Methods

### Participants

Healthy older (*n* = 12, 7 M, age: 66–77 y) and younger adults (*n* = 11, 6 M, age: 20–25 y) participated in the study. In G^*^Power (v.3.1.), we conducted *post hoc* power analysis, considering as input the outcomes of Root Mean Square (RMS) amplitude for muscle main effect (effect size f calculated based on partial eta-squared $\left(\eta_{p}^{2}\right)$ = 1.88; α error = 0.05; total sample size = 23) that resulted in a power (1-β err prob) = 1. Exclusion criteria were self-reported pain in the lower extremity; lower limb musculoskeletal injury or surgery that could affect balance and the ability to perform the STS task; neurological and cardiologic disease, and inability to perform STS task without assistance. The Ethical Committee of the Center for Human Movement Sciences, University Medical Center Groningen, approved the study protocol and the informed consent document (#ECB2017.06.12_1), which all participants signed. The procedures of this study were performed in accordance with the Declaration of Helsinki (World Medical Association Declaration of Helsinki, 2013).

### General Experimental Procedures

**Figure 1:**
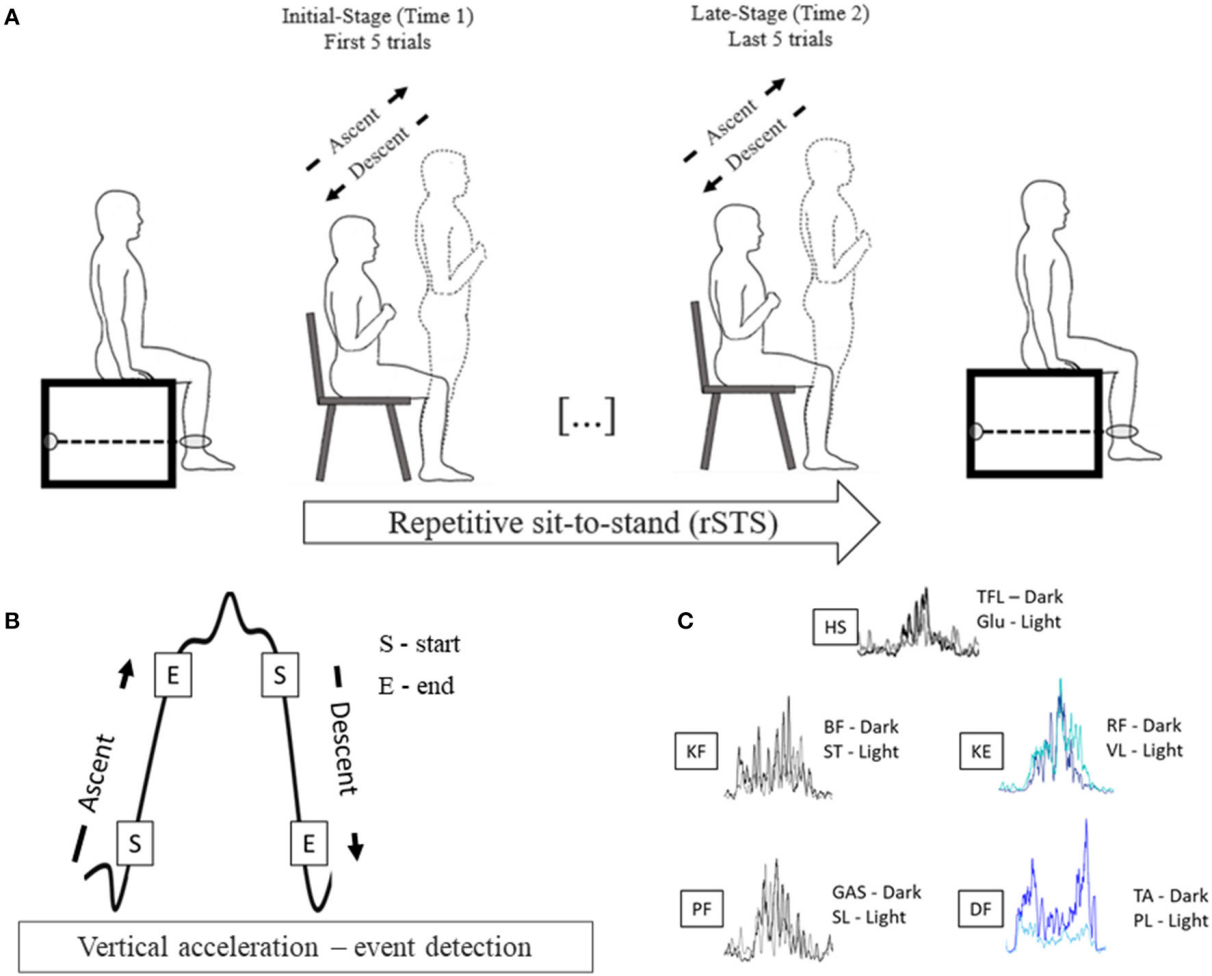
**(A)** Experimental design; **(B)** Vertical acceleration profile of a sit-to-stand bout to exemplify the events used to detect the start and end and determine the sit-to-stand phases; **(C)** Filtered and rectified EMG of one STS bout of one participant showing similar temporal activation for pooling proposes.

Participants visited the laboratory one time and completed the Multidimensional Fatigue Inventory to assess the trait level of fatigue (Smets et al., 1995); performed the Short Physical Performance Battery to characterize mobility, balance, and leg strength (Guralnik et al., 1995), and underwent a procedure to determine voluntary muscle activation, maximal voluntary isometric force (MVIF) in a custom-built dynamometer before (time 1) and after (time 2) rSTS. Participants performed the STS task at metronome-controlled cadence until they could not perform any repetition. During the rSTS bout, surface EMG activity in ten muscles on the dominant leg and vertical acceleration were recorded (Figure 1A).

### MVIF

Participants, seated on a custom-built dynamometer (knee, hip in 90° of flexion), with the non-dominant lower leg strapped (~10 cm superior to lateral malleolus). The non-dominant leg was chosen because we used twitch interpolation (electrical stimulation) for another aspect of this experiment (Santos et al., 2019) that would have damaged the EMG electrodes equipped with a pre-amplifier. Participants were instructed to contract the quadriceps as forcefully as possible and maintain it for 5 s (Santos et al., 2019). After 2 trials of familiarization, participants performed 2 MVIF trials before (separated by 2 min) and one immediately after rSTS. Peak of force was determined as before (highest trial performance) and after rSTS. We avoided more trials because of the uncomfortable situation that twitch interpolation would cause.

### rSTS

Sitting on the front third of the seat of the chair (0.43 m height × 0.41 m width × 0.42 m depth) and leaving the hamstrings EMG sensors free, participants placed their feet in a comfortable position. With the arms crossed at the chest, they stood up to a full extension of the knee and hip joints and sat back down, until touching the seat, to the beat of a metronome set at 30 beats/min (one complete cycle per cycle, 2 s). Participants were instructed to perform the STS movement as long as they could until they were unable to continue. The task was stopped if they reported not being able to continue, performed the task for 30 min, or not being able to keep the cadence of the movement in three sequential trials after specific instruction of waiting for a cycle to synchronize the STS with the metronome. Participants performed no more than five repetitions to become familiar with the task and cadence following for a period of rest (at least 1 min) which we reinforced the instruction regarding the task and scale used to assess the rate of perceived exertion (RPE – detailed below). Duration of the STS bout and the number of complete repetitions were recorded. During the task, two assessors observed, instructed, and incentivized the participants during the task. Trials in each knee and hip were not fully extended were not considered as a valid repetition, and we immediately instructed the participants to fully extended the knee and hip while rising the chair. Before, during each minute, and immediately after rSTS, participants indicated the RPE (6–20 Borg scale) (Borg, 1982). Specifically, considering the Borg Scale, before starting rSTS, we asked the participants to report the level of tiredness they perceive by calling out the numerical value displayed on the Borg Scale. The assessor sat in a chair and held the 10-point scale at eye level in front of the participant for 10 s before the end of each minute and immediately after rSTS.

### EMG and Accelerometry Data Recording

Following SENIAM recommendations for skin preparation and sensor placements (Hermens et al., 2000), the skin was shaved and cleaned with alcohol on the dominant leg (ball kicking) (Burnett et al., 2011; Hurley et al., 2016). Wireless EMG sensors (Trigno System, Delsys Inc., Boston, USA) were placed on the lateral head of the GAS, soleus (SL), TA, peroneus longus (PL), RF, VL, BF long head, semitendinosus (ST), Tensor Fasciae Latae (TFL) and Glu) (see details and figures of placements in http://www.seniam.org/). Sampling frequency was 2.0 kHz. Vertical acceleration was recorded at 148.1 Hz from the acceleration channel of a Trigno sensor (Delsys, Resolution of 16 bits, Bandwidth of 50 Hz) affixed at mid-thigh (RF sensor).

### Data Analysis

Data were analyzed in custom-made MATLAB codes (Version: 2017b; The MathWorks, Inc., Massachusetts, USA). The vertical acceleration signal was low-pass filtered using a 5-Hz 4th order Butterworth filter. Each STS cycle, defined as seat-off to seat-off, comprised the ascent, stand, descent, and sitting phases based on the vertical acceleration signal (Doheny et al., 2013). The start of ascent and descent phases were determined and detected by the algorithm using vertical acceleration thresholds of 0.2 m/s^2^, and the end of the ascent and descent phases were defined using acceleration thresholds of 0.8 m/s^2^ (Doheny et al., 2013)(Figure 1B). All the events (ascent and descent detections) were plotted and visually inspected to guarantee the accuracy of the detection. The initial five STSs were excluded to avoid the adaptation period to the pace of the metronome. Then, the next 5 STSs (initial-stage) and the last 5 STSs (late-stage) were considered for the analyses. Trials with irregular vertical acceleration curves (e.g., trial longer than 3 s) were excluded. For each phase in the initial- and late-stage, we calculated the jerk as a derivative of the acceleration with respect to time (m/s3) and phase duration (s) as time intervals between each phase.

EMG data in each participant and muscle were visually inspected and bandpass filtered (20–450 Hz, 6th order Butterworth filter). Two procedures were used to analyze the RMS mean amplitude and to determine the muscle onset and offset. Firstly, to calculate the RMS amplitude, data were rectified and smoothed using a moving average with a 50 ms window. Secondly, to determine the muscle onset and offset, Teager–Kaiser energy operator (TKEO) was used to facilitate the muscle onset and offset detection (Solnik et al., 2010). TKEO was applied by the formula:

$$\begin{array}{l} \psi \;\left[x\left(n\right)\right]=\;x^{2\left(n\right)}-x\;\left(n+1\right)*\;x\;\left(n-1\right) \end{array}$$

where x is the non-processed, raw EMG data and n is the sample number. After applying TKEO, the data were rectified and low-pass filtered with a cutoff frequency of 50 Hz (2nd order Butterworth filter) (Solnik et al., 2010). In each STS cycle, activation onsets and offsets events were determined relative to seat-off, using a previously described threshold (mean + 15^*^standard deviations of baseline) (Solnik et al., 2010). After, the events were visually inspected to confirm their accuracy. Muscle onset and activation duration, i.e., the time between muscle onset and offset, was calculated relative to seat-off. Non-normalized RMS-amplitude was calculated in ascent and descent phases, and these peaks were averaged for the initial- (Time 1) and late-stages (Time 2). Due to the similarities between muscles functions, i.e., similar temporal behavioral of activation between muscles (e.g., Figure 1C), for the calculation of RMS-amplitude, muscle onset, and muscle activation duration, the muscles were pooled in groups according to their function at the ankle, knee, and hip joints. Therefore, after we had calculated the outcomes for each muscle individually, we pooled 10 muscles (by averaging the individual muscle values for each outcome) into five muscle groups: plantar flexors (GAS and SL), dorsiflexors (TA and PL), knee extensors (RF and VL), knee flexors (BF and ST), and hip stabilizers (TFL and Glu). In addition, the level of co-contraction was calculated between dorsiflexor and plantar flexors and between knee extensors and knee flexors by using co-contraction index (CI) (Falconer and Winter, 1985), quantified as:

$$\begin{array}{l} CI=\left(\frac{2*RMS\;Antagonist}{RMS\;total\;}\right)*100 \end{array}$$

where the antagonist was the muscle group that completed the second action in each phase, and the RMS total was the sum of the RMS amplitude of agonist and antagonistic muscle pairs (Falconer and Winter, 1985).

### Statistical Analysis

Statistics analyses were conducted in SPSS for Windows (Version 25, IBM Corp, Armonk, NY). Group characteristics were normally distributed and compared using a *t*-test. To compare MVIF, and RPE, we ran a repeated-measures ANOVA with as between factor Age (younger vs. older adults) and within factor Time (before rSTS: time 1 vs. after rSTS: time 2). Specifically to examine if Age groups differ in RPE at the initial minutes (first 3) of rSTS, an additional repeated-measures ANOVA was conducted as between factor Age and within factor Minute (Pre vs. min. 1 vs. min. 2 vs. min. 3). Since Shapiro-Wilk revealed a non-normal distribution of EMG data, RMS-amplitude, muscle onset, and muscle activation duration were log-transformed to further analysis. At initial-stage, jerk and phase duration were compared between Age by *T*-test. To compare RMS-amplitude at initial-stage, we conducted repeated-measures ANOVA between Age and within Muscle (plantar flexors vs. dorsiflexors vs. knee flexors vs. knee extensors vs. hip stabilizers) and Phase (ascent vs. descent). Muscle onset and activation duration were compared by repeated-measures ANOVA between Age and within Muscle. Regarding the effects of rSTS, accelerometer data were compared by repeated-measures ANOVA between Age and within Time (initial-stage: time 1 vs. late-stage: time 2). CI was compared by repeated-measures ANOVA between Age and within Phase and Time. We applied repeated-measures ANOVAs (Age vs. Muscle vs. Phase) to compare the magnitude of change (Δ = time 2–time 1) of RMS-amplitude, and between Age and within Muscle to compare Δ of muscle onset and muscle activation. The level of significance adopted was *p* ≤ 0.05. ANOVA effect size was estimated using partial eta squared ($\eta_{p}^{2}$) with $\eta_{p}^{2}$ < 0.25, 0.26–0.63 and >0.64 as small, medium and large effects size (Cohen, 1988). Whether main effects and/or interactions were significant, we set *post hoc* for each factor and the level of significance was adjusted for multiple comparisons by using Bonferroni correction. Additionally for *post hoc* comparisons, Cohen's *d* (*d*) was calculated, and we interpreted 0.21–0.50, 0.51–0.79, and >0.79 as small, medium, and large effect sizes, respectively (Cohen, 1988). For main effects and/or interaction, we reported the relative changes expressed as percentages, where younger, time 1 and ascent values were used as reference values for Age, Time, and Phase differences.

## Results

### Participants and STS Performance

The two age groups were similar in height, body mass, body mass index, mobility, and trait of fatigue level (Table 1). Older vs. younger adults performed on average 467 fewer STS trials (*p* < 0.01) (2 younger adults performed the task for 30 min).

**Table 1:** Participants' characteristics and scores on questionnaires.

	**Older**	**Younger**	***p*-value**
*N* (Male)	12 (7)	11 (6)	–
Age, range (years)	71.00 (66–77)	22.45 (20–25)	<0.01
Height (cm)	173.13 ± 2.22	177.45 ± 2.77	0.17
Body mass (kg)	73.92 ± 3.06	69.82 ± 3.43	0.07
Body mass Index (kg/m^2^)	24.66 ± 1.12	22.17 ± 0.64	0.04
SPPB (scores)	12.00 ± 0.00	12.00 ± 0.00	1.00
MFI (scores)	35.50 ± 2.95	38.18 ± 2.82	0.52
STS (rep)	134.13 ± 29.39	600.9 ± 52.49	<0.01
STS duration (min)	4.47 ± 1.10	20.03 ± 1.75	<0.01

*Values are means and SEs. SPPB, short physical performance battery; MFI, multidimensional fatigue inventory; STS, sit-to-stand*.

### MVIF and RPE

ANOVA main effect and interaction and $\eta_{p}^{2}$ are described in Table 2. ANOVA outcomes for non-significative effects are detailed in Supplementary Material 1.

**Table 2:** ANOVA main effects and interaction and partial eta-squared ($\eta_{p}^{2}$) for the outcomes.

**Outcomes**	**ANOVA effects**	**F _**(df)**_**	***p***	**$\left(\eta_{p}^{2}\right)$**
**Force outcomes and RPE**				
MVIF	Age main effect	8.58 _(1.21)_	<0.01	0.29
	Time main effect	31.66 _(1.21)_	<0.01	0.60
RPE – Pre vs. Post (Borg)	Time main effect	424.27 _(1.21)_	<0.01	0.95
RPE minutes (Borg)	Age main effect	18.91 _(1.21)_	<0.01	0.47
	Minute main effect	86.86 _(3.63)_	<0.01	0.80
	Age*Minute	4.27 _(3.63)_	0.02	0.17
**Initial-stage**				
RMS-amplitude	Muscle main effect	72.30 _(4.84)_	<0.01	0.78
	Age*Muscle	3.40 _(4.84)_	0.01	0.14
	Phase*Muscle	12.27 _(4.84)_	<0.01	0.37
	Age*Phase*Muscle	4.34 _(4.84)_	<0.01	0.17
Activation Onset	Muscle main effect	5.91 _(4.84)_	<0.01	0.22
	Age*Muscle	2.46 _(4.84)_	0.05	0.11
Activation duration	Age main effect	16.07 _(1.21)_	0.01	0.43
	Muscle main effect	8.10 _(4.84)_	<0.01	0.28
**rSTS effects**				
RMS-amplitude	Muscle main effect	6.57 _(4.84)_	<0.01	0.24
Ascent Phase duration	Time main effect	9.95 _(1.21)_	<0.01	0.36
CI – KF/KE Descent	Age main effect	10.08 _(1.21)_	<0.01	0.34
CI – DF/PF Descent	Time main effect	12.69 _(1.21)_	<0.01	0.28
CI – KF/KE Ascent	Time main effect	6.39 _(1.21)_	0.02	0.23

*RPE, Rate of perceived exertion; MVIF, Maximum voluntary isometric force; RMS, Root mean square; rSTS, repetitive sit-to-stand; CI, Co-contraction Index; KF, Knee flexors; KE, Knee extensors; DF, Dorsiflexors; PF, Plantar Flexors*.

**Figure 2:**
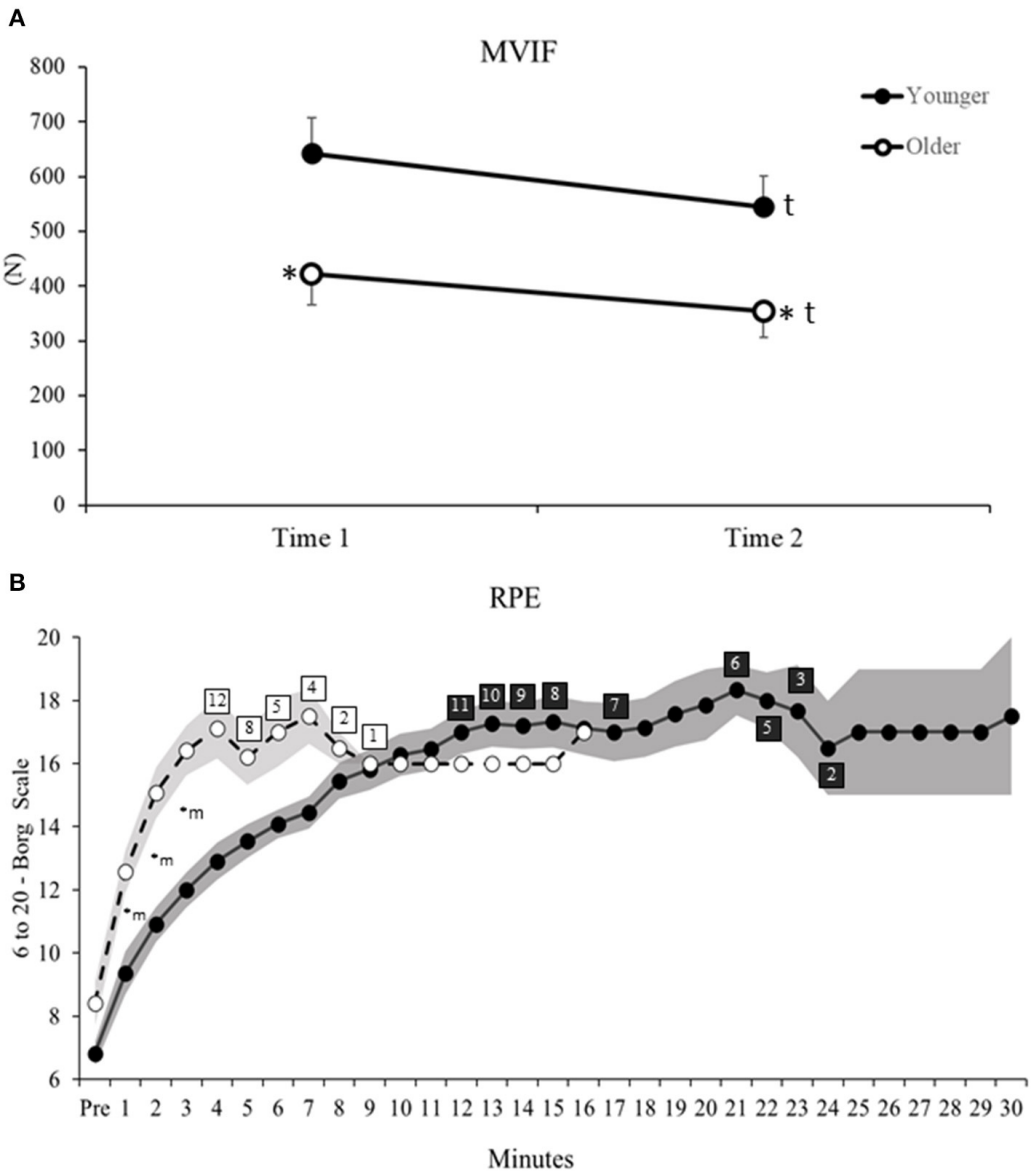
**(A)** Maximum voluntary isometric force (MVIF) before (Time 1) and after (Time 2) rSTS. **(B)** Rate of perceived exertion (RPE) measured by 6–20 Borg scale during each minute of rSTS in younger (filled circles) and older adults (open circles). Shaded areas indicate standard deviation. Numbers on open and filled rectangles indicate the amount of older and younger participants who continued performing rSTS at that time. *Age differences, ^t^Time main effect, ^m^Minutes differences related to Pre (before rSTS).

ANOVA reveled significant Age and Time main effects for MVIF (Table 2). *Post hoc* for Age main effect indicated that older vs. younger adults had 35% lower MVIF (*d* = 1.19, Figure 2A). For Time main effect, after rSTS, MVIF decreased (~16%) in both age groups (*d* = 0.51, Figure 2A).

For Pre vs. Post differences in perceived exertion, ANOVA indicated Time main effect (Table 2). *Post hoc* revealed an increased in RPE in both age groups (before = 7.6 ± 2.1 to after rSTS = 18.7 ± 1.7, *d* = 5.8, Figure 2B). In addition, considering changes in RPE across the first 3 min, ANOVA revealed Age by Minute interaction (Table 2). *Post hoc* indicated that both age groups were similar pre rSTS (*p* > 0.05). However, because of the greater growth rate in RPE in the first 3 min compared to pre rSTS in older (49–95%, *d* range = 1.76–3.11) vs. younger (37–76%, *d* range = 1.40–3.28, all *p* < 0.01), older compared to younger adults reported 35, 38 and 37% higher RPE in the minute 1, 2, and 3 of rSTS, respectively (*d* = 0.83, 1.76 and 1.90, all *p* < 0.01, Figure 2B).

Thus, older and younger adults had similar decreases and increases in force and RPE after rSTS, respectively, despite the older adults generating smaller forces overall. In addition, older compared to younger adults indicated a greater increase in RPE already in the first minute of rSTS.

### Age-Effects on Muscle Activation During STS in the Initial-Stage

Age did not affect jerk and phase duration during ascent and descent (*p* > 0.05, Table 3).

**Table 3:** Jerk and duration of sit-to-stand (STS) phases (ascent, stand, descent, sitting for younger and older adults considering time 1 (initial-stage) and time 2 (late-stage) of repetitive STS.

	**Younger**		**Older**	
	**Time 1**	**Time 2**	**Time 1**	**Time 2**
**Jerk (m/s**^**3**^**)**				
Ascent	1.75 ± 0.11	1.45 ± 0.10	1.94 ± 0.22	1.87 ± 0.44
Stand	0.05 ± 0.06	0.03 ± 0.05	−0.05 ± 0.04	−0.03 ± 0.04
Descent	−1.44 ± 0.10	−1.28 ± 0.07	−1.43 ± 0.15	−1.45 ± 0.18
Sitting	−0.18 ± 0.09	−0.12 ± 0.06	0.01 ± 0.06	−0.05 ± 0.04
**Duration (s)**				
Ascent	0.41 ± 0.02	0.47 ± 0.02^t^	0.37 ± 0.04	0.49 ± 0.06^t^
Stand	0.48 ± 0.06	0.49 ± 0.05	0.58 ± 0.05	0.66 ± 0.06
Descent	0.47 ± 0.02	0.50 ± 0.02	0.46 ± 0.05	0.53 ± 0.05
Sitting	0.70 ± 0.07	0.60 ± 0.08	0.73 ± 0.07	0.61 ± 0.07

*Values are means and SEs. ^t^Time main effect*.

**Figure 3:**
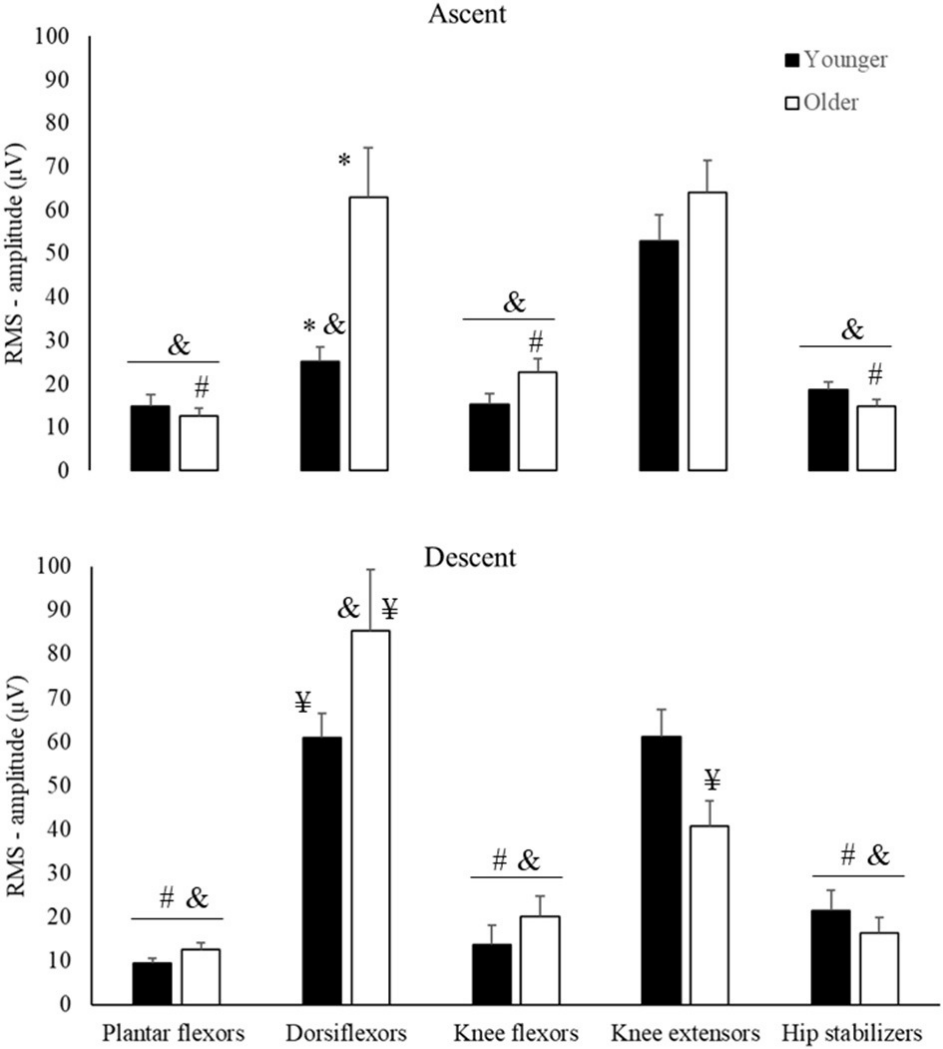
Means and Standard Errors for RMS-amplitude (Y-axis) for younger (filled columns) and older (open columns) adults by muscle groups (horizontal axis) and phases of the sit-to-stand task (Ascent, Descent). *Age differences; ^#^muscles that are different from dorsiflexors; ^&^muscles that are different from knee extensors; ^¥^descent differs from ascent.

There was an Age by Muscle by Phase interaction for RMS-amplitude (Figure 3, Table 2). For Age, *post hoc* comparison revealed that older vs. younger adults rose from the chair with ~60% greater dorsiflexor RMS-amplitude (*d* = 1.16, *p* < 0.01, Figure 3). Concerning phases, *post hoc* comparison indicated that in ascent vs. descent, dorsiflexor RMS-amplitude was ~39% lower (*d* = 0.53) in older adults, but knee extensor amplitude was ~41% higher (*d* = 0.95, all *p* < 0.01) young adults. For muscle comparison, in ascent, whereas older adults had 65–80% higher RMS-amplitude in the dorsiflexors, and knee extensors vs. plantar flexors, knee flexors, and hip stabilizers (*d* range = 1.22–2.64, all *p* < 0.001), youngers' RMS-amplitude of knee extensors was 52–72% higher than dorsiflexors, plantar flexors knee flexors, and hip stabilizers (*d* range = 1.62–2.32, all *p* < 0.01). In descent, both older and younger had 60–90% higher RMS-amplitude in the dorsiflexors and knee extensors than plantar flexors, knee flexors, and hip stabilizers (d range = 1.38–3.11, all *p* < 0.001).

**Figure 4:**
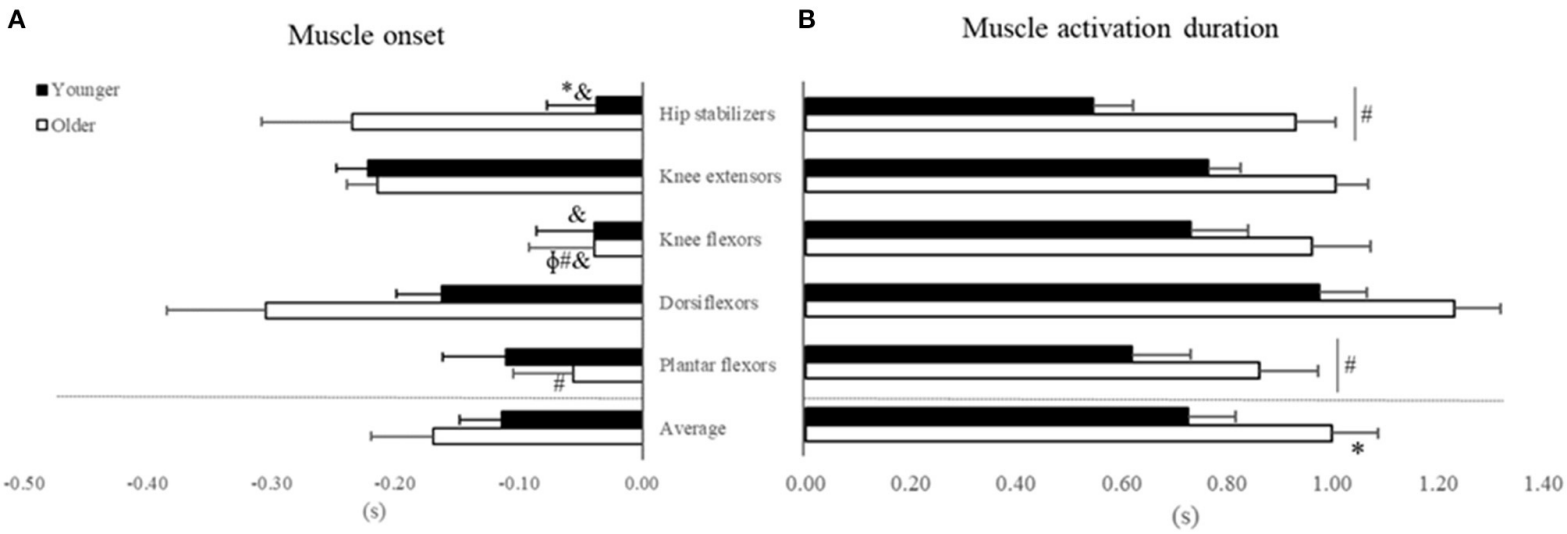
Mean and Standard Error for **(A)** Muscle activation onset and **(B)** muscle activation duration in seconds (X-axis) relative to seat-off (Zero) for younger (filled bars) and older adults (open bars) in the initial-stage. *Age differences; ^#^muscles that are different from dorsiflexors; ^&^muscles that are different from knee extensors; ^ϕ^muscles that are different from hip stabilizers.

ANOVA reveled Age by Muscle interaction for muscle activation onset (Figure 4A, Table 2), but not main effect of Age (*p* > 0.05, Supplementary Material 1). For Age, *post hoc* revealed that older vs. younger activated the hip stabilizers ~80% earlier (*d* = 1.03; *p* < 0.03). For muscle comparison, participants' muscle onsets of dorsiflexors, hip stabilizers, and knee extensors occurred earlier than plantar flexors (42–66%) and knee flexors (73–84%) (*d* range = 1.25–2.09, all *p* < 0.01). Additionally, in younger adults, knee extensors' muscle onset occurred earlier than knee flexors and hip stabilizers (d range = 1.02 and 1.44, *p* < 0.01).

For muscle activation duration (Figure 4B), ANOVA indicated Age and Muscle main effects (Table 2), but not Age by Muscle interaction (Supplementary Material 1). For Age main effect, older vs. younger adults had ~37% (*d* = 1.02) longer muscle activation duration considering all muscles combined (averaged). For muscle main effect, the participants (both groups combined) activated dorsiflexors ~32% longer than the plantar flexors and hip stabilizers (*d* range = 1.12–1.16, all *p* > 0.01).

In overall, our results indicated that older compared to younger adults strongly activated the dorsiflexors to rise from a chair and activated the hip stabilizers earlier and for longer duration the muscles in general. Our results also indicated an age-specific over-activation of dorsiflexor vs. other muscles to rise from a chair. ANOVA outcomes for non-significative effects are detailed in Supplementary Material 1.

### Effects of rSTS on Muscle Activation

ANOVA indicated Time main effect for ascent phase duration (Table 2). However, no Age or Time main effects occurred for jerk and neither interaction for phase duration (all *p* > 0.05, Supplementary Material 1). Time main effect indicated that ascent phase duration became ~20% (*d*: 0.72) longer at the late-stage of rSTS (Table 3).

**Figure 5:**
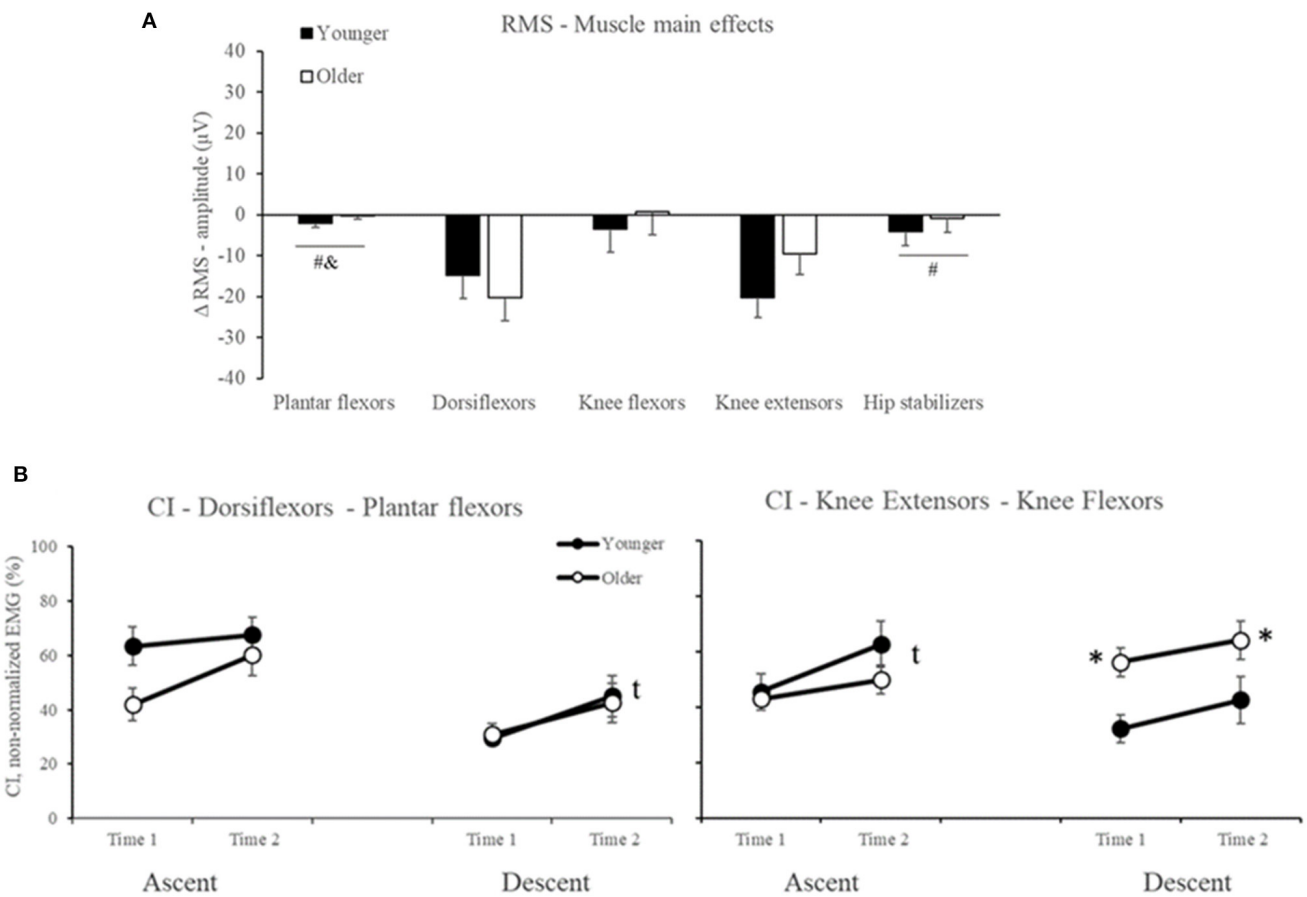
Mean and Standard Error for **(A)** Muscle main effects for the delta score in RMS-amplitude **(B)** co-contraction index (CI) between dorsiflexors and plantar flexors, and between knee extensors and knee flexors in younger (filled symbols) and older (open symbols) adults. *Age differences, ^t^Time main effect. ^#^muscles that are different from dorsiflexors; ^&^muscles that are different from knee extensors.

For RMS-amplitude, ANOVA indicated Muscle main effect (Table 2), but no Age or Age by Time interaction effects occurred (all *p* > 0.05, Supplementary Material 1). For muscle main effects, participants reduced dorsiflexor and knee extensor RMS-amplitude by 25% in the late-stage rSTS compared to no changes in the other muscles (*d* range = 0.85–1.32, all *p* < 0.02, Figure 5A).

For CI, ANOVA indicated Age and Time main effects (Table 2) but not Age by Time interaction (all *p* > 0.05, Supplementary Material 1). For Age main effect, *post hoc* indicated that older vs. younger adults presented 60% higher CI in knee extensors and knee flexors in the descent phase. For Time main effect, *post hoc* to CI between Dorsiflexors and Plantar flexors in descent phase and to Knee extensors and Knee flexors in the ascent phase indicated that both older and younger increased by 45 and 31% (*d* = 0.70 and 0.60) the co-contraction in both muscle groups, respectively (Figure 5B).

In summary, our results indicated that rSTS caused similar reductions in dorsiflexor and knee extensor RMS-amplitude and CI between younger and older adults. No rSTS effects occurred for muscle activation onset and duration (all *p* > 0.05, Supplementary Material 1).

## Discussion

In a partial agreement with the hypothesis, older vs. younger had lower MVIF and greater dorsiflexor activity, longer activation durations of all muscles, and earlier hip stabilizer activity at the initial-stage of rSTS. Older vs. younger completed an average of 467 fewer STSs, accompanied by a quicker increase in RPE. While only two younger adults completed 30 min of the STS task, none of the older adults were able to perform the task for the full 30 min. At the late-stage of rSTS, MVIF and dorsiflexor and knee extensor activation amplitude decreased similarly in both age groups. Thus, older adults, by performing remarkably fewer STS trials, had minimized changes in muscle activation, voluntary force, and motor function. It seems reasonable that had older adults been able to continue performing the task, the effects of rSTS on muscle activation and voluntary force would have been much more pronounced, amplifying age-differences. The data thus suggest that older vs. younger adults stopped performing the rSTS sooner in order to spare the neuromuscular system from failure, which manifested itself in small changes in maximal voluntary force and muscle activation. Such sparing effects might explain the minimal changes observed in gait after rSTS (Hatton et al., 2013; Santos et al., 2019), suggesting a limited scope of the rSTS task as a perturbation model (muscle fatigability) to probe age-effects on muscle adaptation during functional tasks, such as gait and posture.

### Effects of Age on Muscle Activation During STS Before rSTS

We determined the effects of age on STS muscle activation during the initial-stage of the rSTS series. In agreement with previous data, the activation of knee extensors and dorsiflexors was 70–80% higher than other muscle groups (Figure 3), suggesting strong muscle activation during STS (Jeon et al., 2019). As expected, older vs. younger adults rose from the chair with a higher, earlier, and longer muscle activation pattern. The age-specific 65–90% greater knee extensor activation preferentially helps older adults to accelerate the center of mass (CoM) upward (Bryanton and Bilodeau, 2017, 2019; Jeon et al., 2019). Such strong activation of the knee extensors may be needed because MVIF was 35% lower in older than younger adults (Figure 2) and the activation demand can be as high as 90% of the maximum during ascent (Hortobágyi et al., 2003). These data are compatible with the idea that knee extensor function is a strong predictor of STS performance (Bryanton and Bilodeau, 2017; Mentiplay et al., 2020).

A new observation was that dorsiflexor activation amplitude was over two times higher in older vs. younger adults to rise from a chair, which may indicate a strategy to increase stability. The dorsiflexors control the center of pressure (CoP) under the limits of stability by keeping its position close to the CoM and stabilize the ankle joint (Jeon et al., 2019). Age-specific strategy is also reflected by the 80% earlier onset of the hip stabilizers compared to plantar and knee flexors in older adults only (Figure 4A). Because the phase duration of ascent was similar between groups, this early activation in hip stabilizers is indicative of a trunk strategy to older adults keep up with the metronome. We interpret the age-specific 37% longer activation of the five muscle groups combined (Figure 4) as an inability to relax muscles after force generation (Motawar et al., 2016). We conjecture that the lower MVIF, over-activation in dorsiflexor's RMS-amplitude and longer muscle activation duration in older vs. younger are related to the dramatically fewer number of STS trials (Table 1), and indeed an additional correlation confirmed this (*r* range = −0.51 to −0.66, all *p* < 0.01, details at Supplementary Material 2).

While maximal eccentric compared with concentric voluntary force tends to decrease less with age (Vandervoort, 2002), variability during eccentric force generation increases (Hortobagyi et al., 2001). Indeed, we observed that older vs. younger had 40% lower EMG amplitude of the knee extensors and 30% greater activation of the dorsiflexors during descent vs. ascent (Figure 3). This lower activation signifies altered inter-muscle coordination, as there was an age-specific 60% increase in CI (Figure 5B). There is evidence suggesting an age-specific reduction in the ability to generate high levels of mechanical power for prolonged periods (Petrella et al., 2005), agreeing with prolonged muscle activation results (Figure 4B). Such changes in muscle activation are known to increase energy costs (Hortobagyi et al., 2011) and can, at least partially, explain the age-related higher fatigability (quicker increase in RPE and 467 fewer STS trials, Figure 2B) (Stackhouse et al., 2001). We speculate that the preferentially higher muscle activation during the eccentric phase of the STS (Figure 3B) in older compared with younger adults might be related to the over-activation of higher-order brain areas during eccentric contraction in older adults (Yao et al., 2014), which may be related to the age-related faster rise in perceived fatigability (RPE, Figure 2B).

### Effects of rSTS on Muscle Activation

Perhaps the most remarkable finding was that older vs. younger adults performed 467 times fewer STS repetitions. Independent of age, the temporal structure of the STS changed so that the ascent duration became 20% longer without changes in descent duration, resulting in shortened sitting duration (Table 3). In this manner, participants were able to keep up with the tempo dictated by the beat of the metronome.

Against our hypothesis and also in contrast to previous studies (Bryanton and Bilodeau, 2017, 2019), we did not observe increases in muscle activation of the main movers or compensatory shifts in activation to other muscles at the end of rSTS series. In fact, we observed a ~25% decrease in the dorsiflexor and knee extensor activation (Figure 5A). Such reductions in EMG activity in submaximal fatiguing tasks, although unusual, might be the result of a decrease in number, and to a lesser extent, change in the shape of muscle fiber action potentials (Dideriksen et al., 2010, 2011; Wu et al., 2019). Because the MVIF also decreased by 16%, rSTS performance was probably limited by muscle fatigue and reductions in the neural drive. Such reductions possibly forced older adults to perform 467 fewer STS trials. The decrease in knee extensor and dorsiflexor RMS-amplitude explains the ~38% “apparent” increase in knee and ankle muscle co-contraction. The inability to sustain the rSTS longer by older adults is likely due to the rapid increase in RPE (Figure 2B), which represents a threshold for stopping and avoiding neuromuscular failure.

Unlike the reductions after rapid rSTS in the present study, when rSTS was performed 2 times slower in a higher seat (80% of the lower leg lengths), EMG amplitude increased (Bryanton and Bilodeau, 2017). Therefore, the effects of rSTS on EMG amplitude may be velocity- and task-dependent. Faster repetitive contractions increase the mechanical demand on muscle (Petrella et al., 2005), reducing muscle fiber action potential amplitude and increasing the rate of muscle fatigability (Dideriksen et al., 2010). Faster contractions are particularly difficult for older adults due to a selective loss in type 2 muscle fibers, and prolonged relaxation time (Vandervoort, 2002; Petrella et al., 2005). In contrast to our protocol, slower rSTS protocols (Bryanton and Bilodeau, 2017) progressively increased RMS-amplitude first, followed by a decrease due to fatigability later (Dideriksen et al., 2010). A high seat decreases the mechanical load and muscle effort (i.e., ~25% less quadriceps activation) and peak joint moments and angles (30% less at ankle and knee) (Hurley et al., 2016).

The ramifications of the present data point to the potential limitation of rSTS as a perturbation model to explore age-effects on the adaptability of functional tasks (e.g., posture and gait) to fatigability. By performing considerably fewer STS trials, the effects of rSTS on older adults' muscle activation and MVIF were small and similar to younger adults. Indeed, while participants were unable to continue the rSTS series, MVIF of the knee extensors declined only by 10% after 63 STS trials (Hatton et al., 2013) and did not much further (16%) after 135 trials (Santos et al., 2019). The small increase in force loss after twice as many STS trials implies that the knee extensors are spared from becoming dysfunctional. Such sparing effects may be one mechanism underlying the inexplicably trivial changes in gait biomechanics in obstacle crossing tasks after rSTS (Hatton et al., 2013) and the nil changes in older adults' treadmill walking performance (Santos et al., 2019). The substantially fewer STS trials by older vs. younger supports this interpretation because, with the pace fixed, reduction in trials might be a key factor for older adults to have minimized the demand of the rSTS in general and the load on the knee extensors in particular. Consequently, any carry-over of fatigue induced by the rSTS task to the target task, i.e., gait, is minimized, so that walking performance remains unaffected notwithstanding the muscle “failure” in the perturbation rSTS task just a few minutes earlier.

## Limitations and Conclusions

Study limitations include that the two age groups were functionally similar indicated by physical performance tasks, global cognition, and trait of fatigue (Table 1). While the older group represented an unusually healthy segment of older adults, the similarity between the two age groups eliminated functional differences that could have acted as confounders, allowing us to examine a “pure” age-effect. Second, it would have been necessary to concurrently measure metabolic cost/indices with EMG recordings to see if age-differences in muscle activation in the initial and late-stages are functionally relevant. Although we observed age differences in dorsiflexors' RMS-amplitude at initial-stage that agrees with previous findings (Jeon et al., 2021), our results (regarding age comparison at initial-stage, only) should be carefully interpreted due to the lack of normalization. Even though some studies normalized the EMG signal for the sake of between-group comparisons, there is no consensus on this process (Banks et al., 2017; Shojaei and Bazrgari, 2017; Besomi et al., 2020). Actually, the recommendation for the most appropriate normalization procedures is using a matched maximal voluntary contraction (same task/context as the task of interest) (Besomi et al., 2020). Considering the multi-joint and contraction type characteristics of the STS task, matching a maximal voluntary contraction for normalization with similar characteristics of STS may be particularly complicated. This and other types of normalization also have several limitations, such as individual variation in performing maximal voluntary contractions (Besomi et al., 2020). Therefore, a valid and unanswered question is whether different normalization procedures may solve the individual variation during functional tasks, such as STS. In addition, although we selected a standard chair to increase the ecological validity of our data and to facilitate comparison of our results with the literature (Helbostad et al., 2007; Hatton et al., 2013; Barbieri et al., 2014; Santos et al., 2019), performing rSTS in a standard chair might be harder for taller vs. shorter participants. However, shorter individuals might have difficulty with adequate foot placement during the task, but we did not observe such problems. Further studies should consider EMG activities of additional muscles such as those in the trunk and combine these data with kinetic and kinematic analyses. Such analyses would provide mechanistic insights into the nature of the perturbation created by rSTS. Future studies should also examine the relationship between changes in muscle activation during rSTS series and changes in muscle activation in the target tasks, i.e., gait and posture. Such analyses would shed light on the interaction between age and fatigue concerning motor adaptability.

In conclusion, by performing remarkably fewer STS trials, older adults had minimized the potential effects of fatigability on muscle activation, voluntary force, and motor function. Such a sparing effect may explain the minimal changes in gait after rSTS reported in previous studies, suggesting a limited scope of this perturbation model to probe age-effects on muscle adaptation in functional tasks.

## Data Availability Statement

The data set used and analyzed in this study is available from the corresponding author on reasonable request.

## Ethics Statement

The studies involving human participants were reviewed and approved by Ethical Committee of the Center for Human Movement Sciences, University Medical Center Groningen. The patients/participants provided their written informed consent to participate in this study.

## Author Contributions

PS, CL, LG, IZ, FB, and TH conceptualized and designed the study and approved for final publication. PS performed the data acquisition and wrote the first version of the manuscript. PS, CL, and TH set data analysis and data visualization. PS, CL, FB, and TH interpreted the results. CL, IZ, FB, and TH revised the manuscript critically and contributed with important intellectual content. All authors contributed to the article and approved the submitted version.

## Conflict of Interest

The authors declare that the research was conducted in the absence of any commercial or financial relationships that could be construed as a potential conflict of interest.
