# Supplementary material for: Older Compared With Younger Adults Performed 467 Fewer Sit-to-Stand Trials, Accompanied by Small Changes in Muscle Activation and Voluntary Force
Source: Front Aging Neurosci. 2021 Jun 21;13:679282. doi: 10.3389/fnagi.2021.679282 (PMC8276699; doi:10.3389/fnagi.2021.679282)
Supplement: Supplementary file 1 [file Table_1.DOCX]

Supplementary Material 1

**Supplementary Table 1.** ANOVA outcomes for the non-significant main and interaction effects.

| Outcomes | | ANOVA effects | | F _(df*)_ | p | $\eta_{p}^{2}$ |
| --- | --- | --- | --- | --- | --- | --- |
| Force outcomes and Borg | |  |  |  |  |  |
|  | MVIF | Age*Time | | 1.07 | 0.31 | 0.048 |
|  | Borg | Age main effect | | 0.02 | 0.89 | < 0.01 |
| Initial-stage | |  |  |  |  |  |
|  | RMS-amplitude | Age main effect | | 0.59 | 0.45 | 0.03 |
|  |  | Phase main effect | | 0.01 | 0.93 | < 0.01 |
|  |  | Age*Phase | | 1.99 | 0.17 | 0.09 |
|  | Activation Onset | Age main effect | | 3.96 | 0.06 | 0.16 |
|  | Activation duration | Age*Muscle | | 0.45 | 0.77 | 0.02 |
| rSTS effects | |  |  |  |  |  |
|  | Ascent Phase - Jerk | Time main effect | | 0.53 | 0.47 | 0.02 |
|  |  | Age*Time | | 0.87 | 0.36 | 0.04 |
|  | Stand Phase - Jerk | Time main effect | | 0.63 | 0.44 | 0.03 |
|  |  | Age*Time | | 0.65 | 0.43 | 0.03 |
|  | Descent Phase - Jerk | Time main effect | | 0.04 | 0.85 | 0.01 |
|  |  | Age*Time | | 1.07 | 0.31 | 0.05 |
|  | Sitting Phase - Jerk | Time main effect | | 0.13 | 0.72 | 0.01 |
|  |  | Age*Time | | 0.50 | 0.49 | 0.02 |
|  | Ascent Phase duration | Age*Time | | 1.03 | 0.32 | 0.05 |
|  | Stand Phase duration | Time main effect | | 1.03 | 0.32 | 0.05 |
|  |  | Age*Time | | 0.25 | 0.63 | 0.01 |
|  | Descent Phase duration | Time main effect | | 0.22 | 0.64 | 0.01 |
|  |  | Age*Time | | 0.00 | 0.96 | 0.00 |
|  | Sitting Phase duration | Time main effect |  | 1.28 | 0.27 | 0.06 |
|  |  | Age*Time | | 0.12 | 0.73 | 0.01 |
|  |  |  | |  |  |  |
|  | RMS-amplitude | Age main effect | | 2.19 | 0.15 | 0.09 |
|  |  | Phase main effect | | 0.71 | 0.41 | 0.03 |
|  |  | Age*Phase | | 0.71 | 0.41 | 0.03 |
|  |  | Age*Muscle | | 1.05 | 0.39 | 0.05 |
|  |  | Muscle*Phase | | 0.02 | 1.00 | 0.00 |
|  |  | Age*Phase*Muscle | | 0.35 | 0.84 | 0.02 |
|  | CI – KF/KE Descent | Time main effect | | 2.39 | 0.14 | 0.10 |
|  |  | Age*Time | | 0.04 | 0.84 | 0.00 |
|  | CI – DF/PF Descent | Age main effect | | 0.01 | 0.94 | 0.00 |
|  |  | Age*Time | | 0.26 | 0.61 | 0.01 |
|  | CI – KF/KE Ascent | Age main effect | | 3.55 | 0.07 | 0.14 |
|  |  | Age*Time | | 1.63 | 0.22 | 0.07 |
|  | CI – DF/PF Ascent | Age main effect | | 1.57 | 0.22 | 0.07 |
|  |  | Time main effect | | 3.63 | 0.07 | 0.15 |
|  |  | Age*Time | | 1.42 | 0.25 | 0.06 |
|  | Onset | Age main effect | | 0.48 | 0.50 | 0.02 |
|  |  | Muscle main effect | | 0.63 | 0.64 | 0.03 |
|  |  | Age*Muscle | | 2.19 | 0.08 | 0.09 |
|  | Duration | Age main effect | | 1.40 | 0.25 | 0.06 |
|  |  | Muscle main effect | | 2.80 | 0.04 | 0.12 |
|  |  | Age*Muscle | | 1.46 | 0.22 | 0.06 |
| * degree of fredom involving Age, Phase, and Time factors = 1.21 | | | | | | |
| * degree of fredom involving Muscle factors = 4.84 | | | | | | |
