# Supplementary material for: Older Compared With Younger Adults Performed 467 Fewer Sit-to-Stand Trials, Accompanied by Small Changes in Muscle Activation and Voluntary Force
Source: Front Aging Neurosci. 2021 Jun 21;13:679282. doi: 10.3389/fnagi.2021.679282 (PMC8276699; doi:10.3389/fnagi.2021.679282)
Supplement: Supplementary file 2 [file Table_2.DOCX]

Supplementary Material 2

**Supplementary Table 1:** Correlation at initial-stage/baseline. Spearman correlations between the outcomes that indicated significant age differences at the baseline (initial-stage, maximal voluntary isometric force (MVIF), dorsiflexor’ root mean square (RMS)-amplitude (muscle amplitude), Hip stabilizer’ muscle onset, and in overall (by averaging) muscle activation duration) and number of sit-to-stand (STS).

|  |  | MVIF | Dorsiflexor - RMS amplitude | Hip stabilizer - Onset | Overall Muscle duration |
| --- | --- | --- | --- | --- | --- |
| STS trials | r - value | 0.51 | -0.56 | 0.26 | -0.66 |
|  | p - value | **< 0.01** | **< 0.01** | 0.23 | **< 0.01** |

**Supplementary Table 2.** Correlation late-stage/after STS. Spearman correlation between the rate of changes (delta) induced by rSTS the outcomes that indicated significative time differences and STS (MVIF, RMS-amplitude dorsiflexors and knee extensors in both ascent and descent)

| . |  | |  | | RMS-Amplitude | | | | |
| --- | --- | --- | --- | --- | --- | --- | --- | --- | --- |
|  |  | MVIF | | Dorsiflexor - Ascent | | Dorsiflexor - Descent | Knee extensor - Ascent | Knee extensor - Descent |  |
| STS trials | r - value | -0.31 | | 0.00 | | -0.21 | -0.35 | -0.41 |  |
|  | p - value | 0.16 | | 0.99 | | 0.33 | 0.10 | 0.05 |  |
